# Antagonistic Interaction of Selenium and Cadmium in Human Hepatic Cells Through Selenoproteins

**DOI:** 10.3389/fchem.2022.891933

**Published:** 2022-05-25

**Authors:** S. Ramírez-Acosta, R. Uhlírová, F. Navarro, J. L. Gómez-Ariza, T. García-Barrera

**Affiliations:** ^1^ Department of Chemistry, Research Center for Natural Resources, Health and the Environment (RENSMA), Faculty of Experimental Sciences, Campus El Carmen, University of Huelva, Huelva, Spain; ^2^ Faculty of Chemistry, Brno University of Technology, Brno, Czech; ^3^ Research Center for Natural Resources, Health and the Environment (RENSMA), Integrated Sciences, Cell Biology, Faculty of Experimental Sciences, Campus El Carmen, University of Huelva, Huelva, Spain

**Keywords:** cadmium, selenoprotein, HepG2, ICP-QqQ-MS, column switching, isotopic dilution, selenomethionine

## Abstract

Cadmium (Cd) is a highly toxic heavy metal for humans and animals, which is associated with acute hepatotoxicity. Selenium (Se) confers protection against Cd-induced toxicity in cells, diminishing the levels of ROS and increasing the activity of antioxidant selenoproteins such as glutathione peroxidase (GPx). The aim of this study was to evaluate the antagonistic effect of selenomethionine (SeMet) against Cd toxicity in HepG2 cells, through the modulation of selenoproteins. To this end, the cells were cultured in the presence of 100 µM SeMet and 5 μM, 15 µM, and 25 µM CdCl_2_ and a combination of both species for 24 h. At the end of the experiment, cell viability was determined by MTT assay. The total metal content of Cd and Se was analyzed by triple-quadrupole inductively coupled plasma–mass spectrometry (ICP-QqQ-MS). To quantify the concentration of three selenoproteins [GPx, selenoprotein P (SELENOP), and selenoalbumin (SeAlb)] and selenometabolites, an analytical methodology based on column switching and a species-unspecific isotopic dilution approach using two-dimensional size exclusion and affinity chromatography coupled to ICP-QqQ-MS was applied. The co-exposure of SeMet and Cd in HepG2 cells enhanced the cell viability and diminished the Cd accumulation in cells. Se supplementation increased the levels of selenometabolites, GPx, SELENOP, and SeAlb; however, the presence of Cd resulted in a significant diminution of selenometabolites and SELENOP. These results suggested that SeMet may affect the accumulation of Cd in cells, as well as the suppression of selenoprotein synthesis induced by Cd.

## Introduction

Cadmium (Cd) is a highly toxic metal present in the environment as a consequence of natural and anthropogenic processes, causing its entry and accumulation in the food chain (Rigby and Smith, 2020). Cd toxicity depends on the dose, route, and duration of exposure producing numerous disorders in humans, including reproductive failure (Nasiadek et al., 2019) and DNA damage (Jia et al., 2011), and it is classified as a human carcinogen (Chen et al., 2019). Selenium (Se) is an essential trace element in mammals that can be presented in organic species [selenoamino acids such as selenocysteine (SeCys) and selenomethionine (SeMet) and methylated species such as dimethyl selenide (DMSe) or methyl selenol (CH_3_SeH) and Se-containing proteins like selenoalbumin (SeAlb)] and inorganic species (SeO_3_^2−^ and SeO_4_^−2^) (Fairweather-Tait et al., 2010a). The main source of Se comes from food and nutritional supplements (Wang et al., 2016). Se bioavailability depends on many factors, but it is generally attributed to its chemical form. The absorption of all Se species is relatively high, between 70 and 95%, but differs on the source and the Se status of the individual (Finley, 2006). Inorganic species are better absorbed but less retained by the body than the organic forms (Fairweather-Tait et al., 2010b). Food supplements are based on the use of selenium-enriched yeast since they are the main source of SeMet. Most of the Se ingested is used for the synthesis of selenoenzymes, including selenoproteins from families of glutathione peroxidases (GPXs), thioredoxin reductases (TRXRs), and deiodinases (DIOs), which are involved in numerous metabolic processes (Schomburg et al., 2004). Animal studies have revealed the protective role of Se against Cd in the liver and kidney—the most sensitive organs to the toxicity of this element. The positive effect of Se is mainly attributed to selenoproteins (Huang et al., 2012; Boukhzar et al., 2016). *In vitro* models have become an effective alternative to the use of animal experiments and allow the elucidation of the mechanism of action of Se against toxic metals. The Cd/Se interaction has been studied in different cell lines. For example, the results from the SH-SY5Y catecholaminergic neuroblastoma cell line showed that treatment with 10 µM CdCl_2_ and 100 nM sodium selenite (Na_2_SeO_3_) attenuates the changes in terms of oxidative stress and neuronal sprouting caused by Cd (Branca et al., 2018). Ren et al. (2020) reported that Se pretreatment (Na_2_SeO_3_) markedly represses Cd-induced apoptosis in Leydig TM3 cells. In addition, the level of reactive oxygen species (ROS) decreases, and the c-jun N-terminal kinase (JNK) signaling pathway is blocked. On the other hand, in the avian leghorn male hepatoma (LMH) cell line, Se intervention, in the form of Na_2_SeO_3_, inhibited the Cd-induced lactate dehydrogenase (LDH) release and endoplasmic reticulum (ER) stress crosstalk and autophagy by regulating intracellular Ca^2+^ homeostasis (Zhang et al., 2020b). Also, Cd-induced intracellular Ca^2+^ overload was mitigated by the Ca^2+/^calmodulin (CaM)/calmodulin kinase IV (CaMK-IV) signaling pathway (Zhang et al., 2020b).

However, although the interaction of Se and Cd has been previously reported in human cells (Bianga et al., 2014; Marschall et al., 2017), the information about the influence of Cd exposure and Se supplementation on Se metabolites and expression profiles of selenoproteins is limited. To this end, the selenoproteome of hepatic carcinoma cells was quantified after Cd and/or Se exposure by column-switching combining affinity chromatography and size exclusion chromatography coupled to triple-quadrupole inductively coupled plasma–mass spectrometry (ICP-QqQ-MS).

## Materials and Methods

### Preparation of Cadmium and Selenium Solution

Stock standard solutions of CdCl_2_ (Sigma-Aldrich, St. Louis, MO, United States) and SeMet (Sigma-Aldrich, St. Louis, MO, United States) were prepared in deionized water using a Milli-Q system (Millipore, Burlington, MA, United States) at a concentration of 10 mM. This solution was sterilized using a syringe filter with a 0.22 µM pore size and stored in darkness at 4°C. A working solution of 1,000 µM of Cd and SeMet was prepared freshly in culture media before each exposure experiment.

### Cell Culture

The hepatocellular carcinoma (HepG2) cell line was purchased from the European Collection of Cell Cultures (ECACC) (Sigma-Aldrich, St. Louis, MO, United States). Cells were cultured in Dulbecco’s Modified Eagle Medium (DMEM/F12) (Gibco Life Technologies, Grand Island, NY) containing 10% fetal bovine serum (FBS) (Gibco Life Technologies, Grand Island, NY), 1% non-essential amino acids (NEAA) (Gibco Life Technologies, Grand Island, NY), and 1% penicillin-streptomycin (Gibco Life Technologies, Grand Island, NY) and maintained at 37°C in an atmosphere with 5% CO_2_ and 95% relative humidity. The medium was changed every 48 h. Once it reached 80% of confluence, the cells were detached using TrypLE Express (trypsin replacement) (Gibco Life Technologies, Grand Island, NY) and sub-cultured once a week.

### Cytotoxicity Assay

Cell cytotoxicity induced by Cd and SeMet on HepG2 cells was evaluated by MTT [3-(4,5-dimethylthiazol-2 yl)-2,5-diphenyl tetrazolium bromide] (Sigma-Aldrich, St. Louis, MO, United States) assay. Briefly, HepG2 cells were cultured in a 6-well plate at a density of 5 × 10^5^ cells/well and incubated for 24 h. After growth in the cell cycle, cells were exposed for 24 h to increasing Cd and Se concentrations, ranging from 0 to 100 μM, in order to determine their effects on cell viability. At the end of time exposure, the culture medium was removed, and 400 µl of MTT solution (2.5 mg mL^−1^) was added and incubated for another 3 h at 37°C and 5% CO_2_ atmosphere. The MTT solution was discarded, and the reaction product obtained, formazan, is solubilized with sodium dodecyl sulfate (SDS) (Fisher Scientific Co., Nepean, Ontario, Canada) solution (10% p/v). Finally, the absorbance was measured at a wavelength of 540 nm using a Helios Gamma UV-Vis spectrophotometer (Thermo Fisher Scientific, Bremen, Germany). Cell viability is expressed as a percentage relative to the control group. The cells cultured in a standard medium were used as the positive control (100% of viability).

### Cell Exposure

**FIGURE 1:**
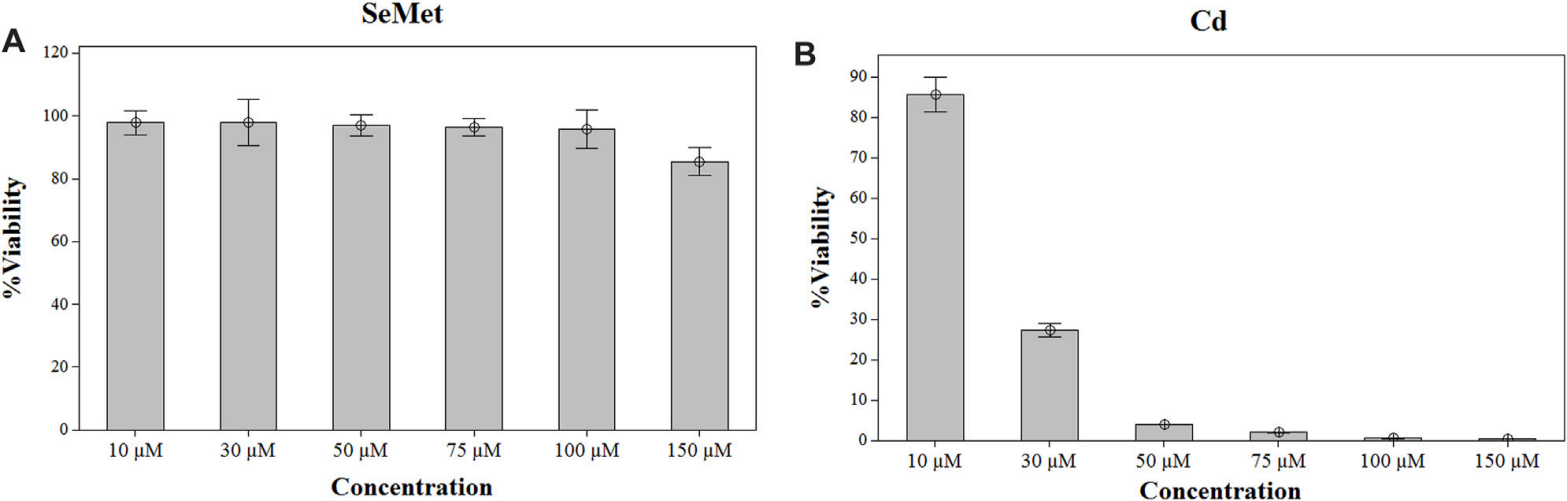
Effect of SeMet **(A)** and Cd **(B)** on cell viability in HepG2 cells. Cells were exposed to the indicated concentrations of SeMet and CdCl_2_ for 24 h.

Cell viability was not affected after selenium exposure in the entire range of concentrations studied (Figure 1). Therefore, a final concentration of 100 µM Se was chosen for the subsequent experiment exposure. In the case of Cd, a significant reduction of > 70% in cell viability was observed at concentrations higher than 30 µM (Figure 1). Consequently, three different Cd concentrations lower than 30 µM were employed. To determine the interactions of these elements in the *in vitro* model of HepG2 cells, they were exposed during 24 h to 100 µM SeMet, alone or in combination with different Cd concentrations, as follows: 1) 0 µM Cd + 0 µM Se (the control group), 2) 100 µM Se (the Se group), 3) non-cytotoxic concentration of 5 µM Cd (the Cd5 group), 4) 5 µM Cd + 100 µM Se (the Cd5+Se group), 5) lowest effective dose of 15 µM (the Cd15 group), 6) 15 µM Cd + 100 µM Se (the Cd15 + Se group), 7) high effective dose of 25 µM Cd (the Cd25 group), and 8) 25 µM Cd + 100 µM Se (the Cd25 + Se group).

At the end of the experiment, cells were washed with phosphate-buffered saline (PBS) (Gibco Life Technologies, Grand Island, NY) three times and then collected by mechanical harvesting using a cell scraper to ensure the integrity of the cells and to avoid chemical interferences with other reagents.

### Determination of Selenium and Cadmium Contents

The total metal content in cell pellets and culture media were analyzed in an ICP-QqQ-MS model Agilent 8800 Triple Quad apparatus (Agilent Technologies, Tokyo, Japan). Harvested cells were previously homogenized with a buffer solution containing 150 mM NaCl (Sigma-Aldrich, St. Louis, MO, United States), 20 mM HEPES (Sigma-Aldrich, St. Louis, MO, United States), 1 mM EDTA (Sigma-Aldrich, St. Louis, MO, United States), 10% glycerol (Sigma-Aldrich, St. Louis, MO, United States), 0.5% Triton X-100 (Sigma-Aldrich, St. Louis, MO, United States), and 1% sodium dodecyl sulfate (Fisher Scientific Co., Nepean, ON, Canada). The cell homogenate was submitted to microwave-assisted acid digestion using a Mars 6 reaction system (CEM Corporation, Matthewa, NC, United States). For this, an aliquot of 100 µl of the cell sample was digested in 490 µl of HNO_3_ (Fisher Scientific Co., Nepean, ON, Canada) and 10 µl of HCl (Fisher Scientific Co., Nepean, ON, Canada). Mineralization was carried out at 200 W from room temperature ramped to 180°C for 30 min and held for 10 min. A second ramp was performed from 200W to 400 W for 20 min and held for 20 min. After digestion, cell samples were 5-fold diluted to achieve a final concentration of 5% HNO_3_ and 100 μg L^−1^ of Rh (internal standard) (Sigma-Aldrich, St. Louis, MO, United States) and filtered through 0.45 µM PTFE syringe filters.

For the analysis of culture media, an aliquot of 1 ml of the sample was collected and diluted using the same procedure as cell samples. The ICP-QqQ-MS operational conditions are shown in Supplementary Table S1. The certified reference material BCR-274 Single Cell Protein (Sigma-Aldrich, St. Louis, MO, United States) was used to validate the methodology (Supplementary Table S2).

### Selenoprotein Speciation

Selenoproteins were extracted from HepG2 cells using the CelLytic™ MT extraction reagent (Sigma-Aldrich, St. Louis, MO, United States), following the manufacturer’s instructions with some brief modifications. A cell pellet of 15 F0B4·10^6^ cells was lysed with 100 µl of CelLytic™ MT containing protease inhibitor cocktail (Sigma-Aldrich, St. Louis, MO, United States)) in a shaker for 15 min. Then, the lysed cells were centrifuged at 15,500 *g* for 10 min at 4°C, and the supernatant was collected for the subsequent analysis. The chromatographic separation of the selenoproteins (GPx, SELENOP, and SeAlb) was performed, as described elsewhere (Callejón-Leblic et al., 2018), using the column switching method that allows the simultaneous separation of selenoproteins and selenometabolites. The separation consists of two 5-ml HiTrap^®^ desalting columns (GE Healthcare, Uppsala, Sweden) connected in series with a 1-ml heparin-sepharose (HEP-HP) column (GE Healthcare, Uppsala, Sweden) and a 1-ml blue-sepharose (BLUE-HP) column (GE Healthcare, Uppsala, Sweden) by ultra-high performance liquid chromatography (model 1,260 Infinity Quaternary LC, Agilent Technologies, Tokyo, Japan). The absolute quantification of selenoproteins was carried out in an ICP-QqQ-MS model Agilent 8800 Triple Quad apparatus (Agilent Technologies, Tokyo, Japan), employing the conditions from Supplementary Table S1. For the isotope dilution analysis, ^74^Se (Cambridge Isotope Laboratories, Andover, MA, United States) was also introduced *via* T-connector into the system.

### Statistical Analysis

Statistical analyses were performed by Minitab 21 Statistical Software (State College, PA, United States). The results are expressed as means ± SD of at least three replicates of each group. All experiments were repeated three times. The Anderson–Darling normality test was used to determine whether data are not normally distributed. Differences between groups were tested using the Kruskal–Wallis test (no normal distribution) and ANOVA (normal distribution). The level of *p* < 0.05 was considered statistically significant.

## Results

### Effects of CdCl_2_ and SeMet on Cell Viability

To select the dosage for the exposure, HepG2 cells were treated with a range of concentrations from 0 to 150 µM of SeMet and CdC_2_ for 24 h. After the exposure, cell viability was measured by the MTT method. Selenomethionine exposure did not affect the cell viability of HepG2 cells in the range of concentrations from 10 to 100 μM, but a slight decrease was noticed at a concentration of 150 µM (Figure 1). Therefore, a final concentration of 100 µM SeMet was chosen for the subsequent experiment exposure. In the case of Cd, a reduction of >15% in cell viability was observed at concentrations higher than 10 µM (Figure 1), and at a concentration of 30 µM of CdCl_2_, a significant cell reduction of 70% was detected. Consequently, three different concentrations of CdCl_2_ were used (5, 15, and 25 µM).

**FIGURE 2:**
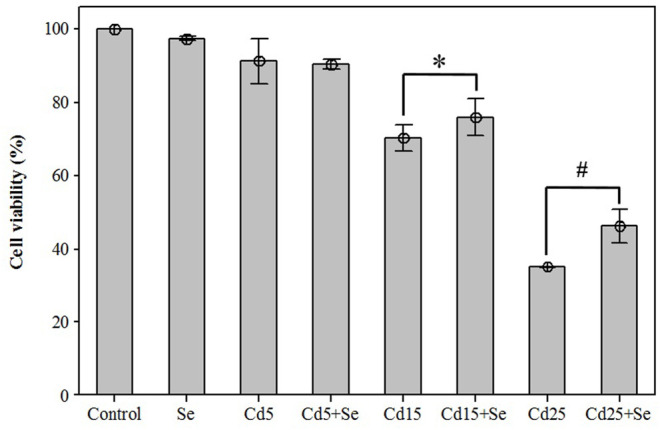
Effects of different concentrations of CdCl_2_ and/in combination with SeMet on cell viability in HepG2 cells. Values are expressed as mean ± S.D. (*n* = 3) (*): significant differences between groups Cd15 and Cd15+Se; (#): significant differences between groups Cd25 and Cd25+Se.

To evaluate the effects on cell viability of the selected CdCl_2_ concentrations alone and in combination with SeMet, a new MTT assay was carried out. The results are presented in Figure 2. The lowest concentration of CdCl_2_ (Cd5 group) reduced cell viability to 91.19 ± 2.50%, but the combination with SeMet (Cd5+Se group) did not significantly affect cell viability (*p* = 0.591). The result for the Cd15 group was 70.27 ± 1.44%, and the presence of SeMet (Cd15 + Se) increased the cell survival to 75.82 ± 2.02% (*p* = 0.018). In the Cd25+Se group, a very significant increase to 46.01 ± 1.89% compared to the Cd25 group (35.04 ± 0.09%) was detected. Our results showed that Cd hepatotoxicity could be mitigated by SeMet supplementation.

### Cadmium and Selenium Concentrations in Hepatocellular Carcinoma Cells and Culture Media

The analysis of Se and Cd by ICP-QqQ-MS in cell pellets and culture media is summarized in (Table 1). The detection limits obtained for the analytical procedure (LOD) were 0.008 ng g^−1^ for Se and 0.001 ng g^−1^ for Cd. The basal concentration (control group) of Se in HepG2 cells is 0.052 µM, while the concentration of Cd was <LOD. In the culture medium, the concentrations of both elements were <LOD. The groups not exposed to SeMet resulted in similar concentrations in all of them, varying in the range of 0.032–0.041 µM. The addition of SeMet in the culture medium resulted in an uptake by HepG2 cells ranging between 11.02 and 15.494 µM. The concentrations of Cd in the Cd5 and Cd5+Se groups were 3.851 µM and 3.916 µM, respectively, and no statistically significant differences were observed (Table 2). This assumes an absorption of approximately 78% of the exposed Cd by the cells. However, the cells exposed to a concentration of 15 µM of Cd absorbed between 56 and 58% of the exposed Cd, showing a slight but significant increase in the Cd15 + Se group (*p* = 0.014). At the maximum concentration, 25 μM Cd, HepG2 cells only absorbed between 34 and 36% of the total Cd. The total Cd content in the Cd25 + Se group was slightly high (*p* = 0.02).

**TABLE 1:** Total Se and Cd contents (µM) in cell pellets and culture media expressed as mean ± standard deviation (*n* = 3) and Cd and Se absorption percentage (%).

Group	(Se) (µM)		Absorption	(Cd) (µM)		Absorption
	Cells	Culture media	Se (%)	Cells	Culture media	Cd (%)
Control	0.052 ± 0.010	<LOD	—	<LOD	<LOD	—
Se	15.630 ± 0.157	75.559 ± 1.296	15.6	<LOD	<LOD	—
Cd5	0.033 ± 0.003	<LOD	—	3.851 ± 0.006	0.304 ± 0.012	77
Cd5 + Se	15.493 ± 0.220	82.040 ± 0.180	15.5	3.916 ± 0.061	0.294 ± 0.17	78.3
Cd15	0.032 ± 0.006	<LOD	—	8.404 ± 0.045	5.869 ± 0.173	56
Cd15 + Se	13.058 ± 0.376	90.565 ± 0.882	13.1	8.799 ± 0.316	6.451 ± 2.815	58.7
Cd25	0.041 ± 0.005	<LOD	—	8.569 ± 0.002	15.594 ± 0.424	34.3
Cd25 + Se	11.020 ± 0.311	84.138 ± 1.382	11	8.999 ± 0.199	15.694 ± 0.296	36

**TABLE 2:** Concentration of selenoproteins and selenometabolites in HepG2 cells.

Group	GPx (µg Se/g)	Se metabolites (µg Se/g)	SELENOP (µg Se/g)	SeAlb (µg Se/g)
Control	0.010 ± 0.002	0.022 ± 0.002	0.03 ± 0.001	0.004 ± 0.0005
Se	20.495 ± 2.067	16.378 ± 3.995	25.200 ± 0.065	3.041 ± 0.002
Cd5	0.010 ± 0.005	0.168 ± 0.003	0.043 ± 0.001	0.004 ± 0.0004
Cd5+Se	14.120 ± 0.827	4.889 ± 1.243	21.642 ± 1.085	2.6647 ± 0.8357
Cd15	0.019 ± 0.006	0.069 ± 0.001	0.028 ± 0.002	0.005 ± 0.001
Cd15+Se	10.392 ± 1.802	1.773 ± 0.351	13.405 ± 0.400	1.494 ± 0.100
Cd25	0.010 ± 0.001	0.035 ± 0.002	0.025 ± 0.001	0.004 ± 0.001
Cd25+Se	12.392 ± 0.150	2.269 ± 0.291	15.665 ± 0.894	2.749 ± 0.265

### Selenoprotein Speciation

**FIGURE 3:**
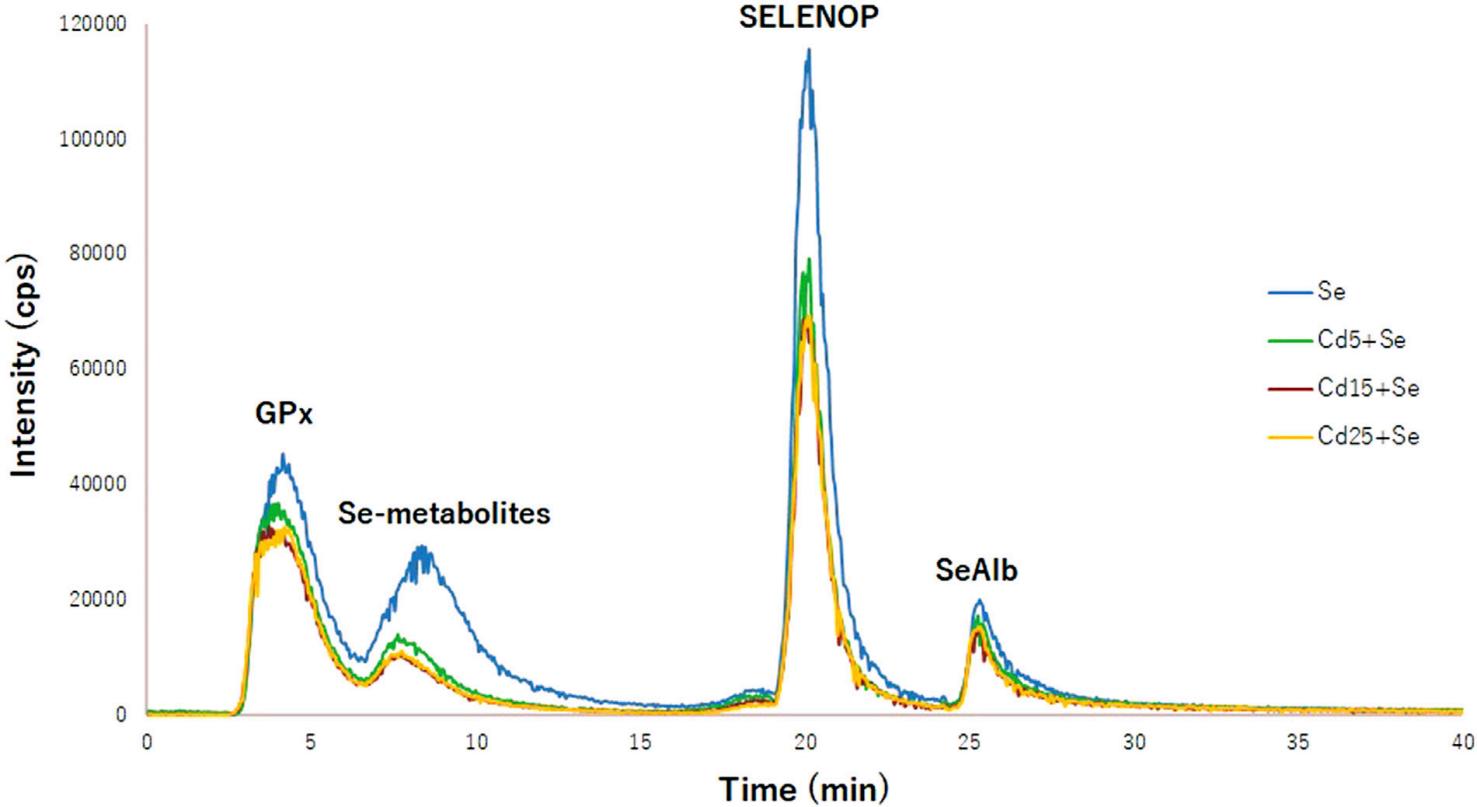
Chromatogram of selenoproteins obtained by 2D-HPLC-SEC-AF-SUID-ICP-QqQ-MS. Blue (Se group); green (Cd5+Se group); red (Cd15+Se group); yellow (Cd25+Se group).

To elucidate whether Cd interferes with selenoprotein synthesis, the concentration of selenometabolites and selenoproteins (GPx, SELENOP, and SeAlb) has been quantified using column switching and a species-unspecific isotopic dilution approach. Figure 3 shows the typical chromatogram for the Se, Cd5+Se, Cd15+Se, and Cd25+Se groups. The relative concentration of the selenoproteins and selenospecies in HepG2 cells is SELENOP > GPx > Se-metabolites > SeAlb. Table 2 summarizes the results obtained from the quantification of selenoproteins and selenometabolites for the different study groups. The results of the statistical analysis are included in Table 3.

**TABLE 3:** *p*-values obtained for each comparison. Significance level *p* < 0.05.

	Selenoproteins and Selenoespecies (µg Se/g)			
Comparison	GPx	Se metabolites	SELENOP	SeAlb
Se *vs*. control	0.007	0.002	0.000	0.000
Cd5 *vs*. control	0.942	0.000	0.000	0.752
Cd15*vs*. control	0.062	0.000	0.479	0.486
Cd25*vs*. control	0.909	0.001	0.094	0.656
Cd5+Se *vs*. Se	0.196	0.009	0.005	0.478
Cd15+Se *vs*. Se	0.074	0.003	0.000	0.000
Cd25+Se *vs*. Se	0.118	0.004	0.000	0.129

As can be observed in Table 2, the concentrations of GPx, selenometabolites, SELENOP, and SeAlb in control HepG2 cells are 0.010, 0.022, 0.03, and 0.004 µg Se/g, respectively. After exposure for 24 h with SeMet, the concentration of the species increases significantly (Table 3), reaching values of 20,495 µg Se/g for GPx, 16,378 µg Se/g for selenometabolites, 25,200 µg Se/g for SELENOP, and 3.041 µg Se/g for SeAlb.

The concentration of selenometabolites was also slightly increased in cells cultured with different doses of Cd. Regarding selenoproteins, only the SELENOP concentration increased in cells that were exposed to 5 μM Cd but not at higher concentrations (15 µM and 25 µM). The co-exposure of SeMet and Cd provokes a decrease in the concentration of GPx in all study groups. However, no statistically significant changes are observed (Table 3). The concentration of selenometabolites in the Se group was 16.378 µg Se/g. Cd exposure significantly decreases the concentration to values of 4.889 µg Se/g (Cd5+Se), 1.773 µg Se/g (Cd15+Se), and 2.269 µg Se/g (Cd25+Se). The SELENOP concentration was also decreased to values of 21.642 µg Se/g (Cd5+Se), 13.405 µg Se/g (Cd15+Se), and 15.665 µg Se/g (Cd25+Se), with respect to the value of the Se group (25.200 µg Se/g). SeAlb concentration was only diminished in cells from the Cd15+Se group.

## Discussion

The results obtained from this study indicated that intracellular Cd concentrations of the groups Cd15+Se and Cd25+Se were significantly lower than those exposed to Cd, and the total Se content was also decreased. Frisk et al. (2002) reported a similar study using K-562 cells that were either pre-treated or simultaneously treated with selenite or selenomethionine (5 or 50 µM) and cadmium nitrate (60 or 75 µM). Their results showed that the pre-treatment with Se caused a lower concentration of Cd in the cells when they were exposed to the higher concentration of Cd (75 µM) but not at the lower concentration (60 µM). On the other hand, the simultaneous treatment of both elements caused an increase in the intracellular Cd at all concentrations, suggesting that Se may affect Cd transport to cells.

In mammals, depending on the bioavailability of Cd, it can behave like other essential elements and can be better assimilated, inducing an imbalance in the normal balance of trace elements and destroying the structure and function of cells (Martelli et al., 2006). Our results indicated that the Cd content was reduced, pointing out that the interaction between Se and Cd produced a Cd-Se complex that affects the composition of these metals in the hepatocyte and presumably in the liver since this same effect has been seen in the liver of rabbits exposed to Cd and Se (Zhang et al., 2021); however, the opposite effect has been observed in mice that were exposed to low and high levels of Se simultaneously with Cd, resulting in a higher Cd concentration in the liver (Rodríguez-Moro et al., 2020).

On the other hand, the effects ofCd-induced hepatotoxicity in birds have also been quantified (Li et al., 2013). Results showed that dietary selenite decreased Cd accumulation and improved antioxidant defense, which attenuated adverse Cd-mediated morphological changes and oxidative stress in liver tissue (Li et al., 2013).

The protective role of Se has been demonstrated by restoring hepatic blood markers such as alanine transaminase (ALT), aspartate transaminase (AST), gamma-glutamyl transferase (GGT), and total bilirubin, (Renugadevi and Prabu, 2010; Abu-El-Zahab et al., 2019; Wang et al., 2020) and antioxidant enzymes such as catalase (CAT) and glutathione peroxidase (GPx) (Ognjanović et al., 1995; Štajn et al., 1997; Jihen et al., 2009); the mechanisms by which trace metal Se suppresses Cd hepatotoxicity in mammals remain unclear.

Regarding selenoprotein concentration, the Se absorption efficiency in HepG2 cells depends on its chemical form, where organic species are more easily transported and assimilated than inorganic species (Wang et al., 2016; Takahashi et al., 2017). The results obtained from this study indicated that the concentration of GPx was not affected by the presence of Cd compared to control cells, but there was a slight decrease in the cells cultured with SeMet. GPx is employed as an oxidative biomarker as its activity is generally induced in response to mild oxidative stress as a defense mechanism. However, a rapid increase in intracellular ROS generation can overwhelm antioxidant mechanisms, resulting in a suppression of antioxidant enzymes (Cozzari et al., 2015).

As seen in both *in vivo* and *in vitro* studies, Se supplementation and exposure results in increased activity, expression, and concentration of most selenoproteins (Stoedter et al., 2010; Zwolak and Zaporowska, 2012; Nelson et al., 2016; Sun et al., 2020; Liu et al., 2021). In liver cells, the expressions of SELENOP and GPx3 exposed to SeMet, MeSeCys, and Na_2_SeO_3_ were increased compared to control cells (Takahashi et al., 2017). An increase in the expression of other selenoproteins such as GPx1 and SELENOK has also been correlated with a higher concentration of sodium selenite (Rusolo et al., 2013). In the study by Jamwal et al. (2016), the GPx activity was significantly (∼35%) decreased in trout hepatocytes treated with Cd alone or in combination with a high concentration of selenite or SeMet (250 µM) relative to control cells. However, a partial recovery of the GPx activity was recorded in hepatocytes exposed to 100 µM Cd in combination with 25 µM selenite or SeMet.

The expression of 24 selenoproteins was determined in chicken hepatocytes that were cultured for 24 h with 5 μM Cd, 1 µM Se, and a mixture of both concentrations (Zhang et al., 2020a). The results presented that 18 of 24 selenoprotein transcripts (GPX1, GPX2, GPX3, GPX4, SelT, Sel15, SelP1, SelP2, TXNRD2, TXNRD3, DIO2, DIO3, SelS, SelK, SelN, SelO, SelU, and SelH) were over-upregulated, and SelW, TXNRD1, and SelX transcripts were downregulated in the Se group compared to the control group. When cells were cultured with Cd and Se, only nine transcripts were overexpressed (GPX1, SelT, SelP1, SelP2, SelS, SelK, SelN, SelO, and SelM), and two were decreased (SelW and SelU) when compared with the group only exposed to Cd (Zhang et al., 2020a).

Selenoproteins may be involved in the antagonistic process against Cd toxicity. In kidney and testicular tissues, it has been seen that Se protected against Cd toxicity by increasing the expression of most of the selenoproteins, including SELENOP and GPX (Messaoudi et al., 2010; Lin et al., 2018). However, our results showed a decrease in the concentration of selenoproteins and selenometabolites when cells are exposed to Cd and Se simultaneously. The recent study by Schwarz et al. (2020) has provided a new perspective on the behavior of selenoproteins in the presence of other metals. In his study, HepG2 cells were exposed to variable concentrations of Cu and Se, revealing that Cu suppresses the mRNA levels of the selenoproteins GPX1 and SELENOW and decreases the activities of GPX and TXNRD. The effects of Cu were inhibited by applying copper chelators, showing that this element interferes with the synthesis and activity of selenoproteins through the limitation of UGA coding (Schwarz et al., 2020). The suppression of selenoprotein synthesis by Cd may also be due to the formation of the above-mentioned Cd-Se complex (Zhang et al., 2021).

Finally, SeAlb has been quantified for the first time in HepG2 cells. SeAlb is a Se transporter protein that is formed in the serum by the replacement of methionine with selenomethionine and transferred to the liver for the synthesis of selenoproteins. The concentration of SeAlb was higher in all Se-supplemented groups. Comparable results were reported from liver cytosolic extracts of mice exposed to Hg and Se simultaneously (García-Sevillano et al., 2015).

## Conclusion

We can conclude that our study has provided new insights into the mechanisms behind the antagonistic interactions between Se against Cd. Se protects HepG2 cells by increasing their cell viability and inhibiting the transport of Cd into cells. The concentration of selenoproteins is also affected by the presence of Cd, interfering with its synthesis and resulting in a lower concentration of selenoproteins. For a better understanding of the Cd-Se interaction in HepG2 cells, it will be necessary to address new studies involving the study of selenometabolites, markers of oxidative stress, and the quantification and characterization of other selenoproteins and the Cd-Se complex.

## Data Availability

The original contributions presented in the study are included in the article/Supplementary Material, further inquiries can be directed to the corresponding authors.
