# Supplementary material for: Antagonistic Interaction of Selenium and Cadmium in Human Hepatic Cells Through Selenoproteins
Source: Front Chem. 2022 May 25;10:891933. doi: 10.3389/fchem.2022.891933 (PMC9174642; doi:10.3389/fchem.2022.891933)
Supplement: Supplementary file 1 [file DataSheet1.docx]

SUPPLEMENTARY MATERIAL

Antagonistic interaction of selenium and cadmium in human hepatic cells through selenoproteins

**S. Ramírez-Acosta^1^, R. Uhlírová^2^, F. Navarro^3*^, J.L. Gómez-Ariza^1^, T. García-Barrera^1*^**

^1^Research Center for Natural Resources, Health and the Environment (RENSMA). Department of Chemistry, Faculty of Experimental Sciences, Campus El Carmen, University of Huelva, Fuerzas Armadas Ave., 21007, Huelva, Spain.

^2^Brno University of Technology, Faculty of Chemistry, Faculty of Chemistry, Purkynova 118, 612 00 Brno, Czech Republic.

^3^Research Center for Natural Resources, Health and the Environment (RENSMA). Integrated Sciences, Cell Biology, Faculty of Experimental Sciences, Campus El Carmen, University of Huelva, Fuerzas Armadas Ave., 21007, Huelva, Spain.

**TABLES**

| **ICP-MS operational conditions** | | **Chromatographic conditions** | |
| --- | --- | --- | --- |
| Forward power | 1550 W | Flow rate | 1.3 mL·min^-1^ |
| Nebulizer | MicroMist | Mobile phase A | 0.05 M ammonium acetate |
| Plasma gas flow rate | 15 L·min^-1^ | Mobile phase B | 1.5 M ammonium acetate |
| Carrier gas flow rate | 1.08 L·min^-1^ | Gradient | 0-12 min 0% B |
| Reaction gases | 40% O_2_, H_2_ |  | 12-25 min 100% B |
| Sampling and skimmer cones | Ni |  | 25-40 min 0%B |
| Sampling depth | 10 mm | Valve position | position 1 (0-20 min) |
| Isotopes monitored | ^74^Se, ^76^Se, ^77^Se, ^78^Se, ^80^Se ^103^Rh, ^111^Cd |  | position 2 (20-24 min) |
| Integration time | 0.3 s |  | position 1 (24-40 min) |

**Table S1.** Operational conditions for speciation of selenoproteins and total metal content.

| **BCR-274 Single Cell Protein certified reference material** | | |
| --- | --- | --- |
| **Element** | ***Obtained value (µg/g)*** | ***Certified value (µg/g)*** |
| Se | 1.07 ± 0.04 | 1.03 ± 0.05 |
| Cd | 0.031 ± 0.004 | 0.03 ± 0.002 |

**Table S2.** Certified and obtained values of BCR-274 reference material using the methodology proposed.

|  | **Cd total content (µM)** | | **Se total content (µM)** | |
| --- | --- | --- | --- | --- |
| **Comparison** | *Cells* | *Culture media* | *Cells* | *Culture media* |
| **Se *vs* Control** | - | - | 0.000 | 0.000 |
| **Cd5+Se *vs* Cd5** | 0.243 | 0.268 | 0.430 | 0.001 |
| **Cd15+Se *vs* Cd15** | 0.014 | 0.005 | 0.000 | 0.000 |
| **Cd25+Se *vs* Cd25** | 0.020 | 0.878 | 0.000 | 0.002 |

**Table S3**. *p*-values ​​for the different comparisons of total Cd and Se content in HepG2 cells and culture media.
